# Supplementary material for: Single-cell genomics highlight MYC-associated metabolic activation and altered cell interactions in T-prolymphocytic leukemia progression
Source: Nat Commun. 2026 Mar 9;17:2319. doi: 10.1038/s41467-026-70185-w (PMC12976252; doi:10.1038/s41467-026-70185-w)
Supplement: Supplementary file 1 — Supplementary Information [file 41467_2026_70185_MOESM1_ESM.pdf]

SUPPLEMENTAL DATA

Supplemental Figures

Supplemental Figure 1

(A) Validation of scRNA sequencing data in patient pair 3 using qRT-PCR. Expression of n=8 deregulated genes was assessed using scRNA sequencing and qRT-PCR. Log2 fold-changes between the active and indolent sample of patient pair 3 displayed strong coherence between both methods (r=0.95, p=0.0003, Pearson correlation, two-sided, grey band: 95% confidence interval). **(B)** t-SNE representation of T-PLL samples and external healthy controls[1] prior to batch correction using Harmony.[2] Subpanels illustrate the distribution of different biological and technical parameters. Healthy-donor derived PBMCs show a distinct clustering from T-PLL samples. T-PLL cells reveal strong patient-specific clustering, while non-tumor cells of T-PLL samples show cell type specific orientation in t-SNE space. **(C)** Correlation matrix of T-PLL cell subcluster gene expression profiles. Spearman rho values are color-coded. Subclusters were calculated for each sample separately using Seurat's FindClusters function at a resolution of 1.[3] Pseudo-bulk aggregated gene expression was used for correlation analysis. Clustering of correlation parameters was performed using Ward's method on Euclidean distances. **(D)** Top panel: Identified clusters as used for cell type identification. Clusters were derived using Seurat's shared nearest neighbor (SNN) modularity optimization at a resolution of 1.5 on batch-corrected PCA space. Bottom panel: Stacked bar plot representing the relative contribution of T-PLL patients and healthy controls to each identified cell cluster. Bars are colored by individual patients, with some clusters displaying patient-specific enrichment, while clusters corresponding to non-tumor cell populations include contributions from multiple patients. Clusters are ordered by cellular composition diversity, from those with mixed contributions across individuals (left) to those dominated by a single patient (right). Source data and complete summaries of statistical analyses are provided in the Source Data file.

Supplemental Figure 1

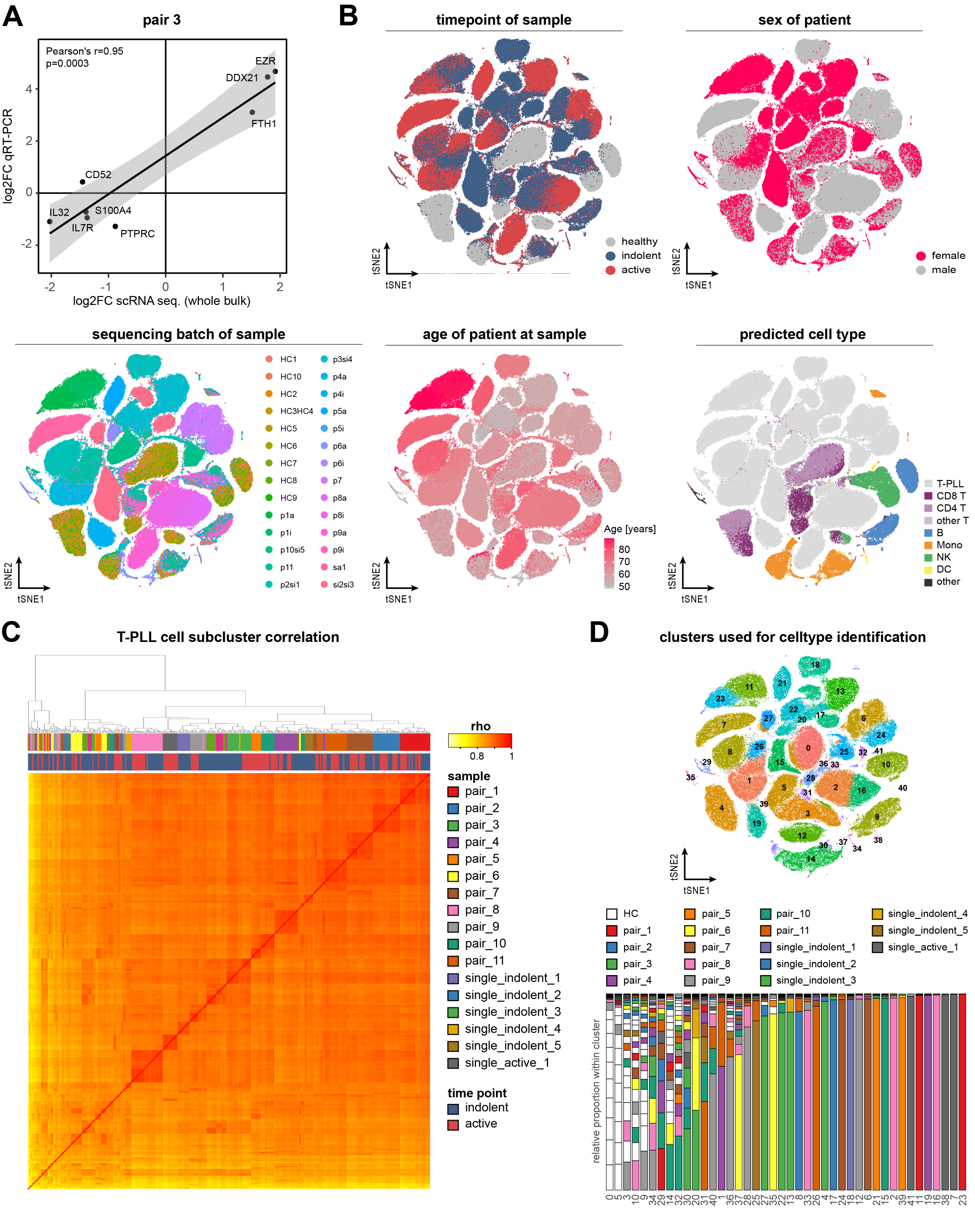

Supplemental Figure 2

(A) Heatmap of top 100 DEGs between active sample T-PLL cells and healthy-donor derived T cells. Colors indicate z-scores of mean expression values per sample (blue: negative z-score, red: positive z-score). DEGs were calculated on pseudo-bulk expression matrices using sex as confounder due to an observed influence of sex on the first principal components of gene expression data. The top 100 DEGs with lowest p-values were selected. **(B-C)** Comparison of differential gene expression data sets in T-PLL. DEGs derived from scRNA sequencing comparing active-disease T-PLL cells (B) and active-disease PBMCs (C) with healthy-donor derived T cells respectively. **(B)** scRNA sequencing-derived log2 fold-changes were correlated with log2 fold-changes derived from a publicly available gene expression array data set[4] (upper panel, 10 healthy-donor CD3+ T cells vs 69 T-PLL samples, rho=0.7592, p<0.0001, Spearman correlation, two-sided) and an mRNA sequencing data set[5] (lower panel, 6 healthy-donor derived CD3+ T cells vs 48 T-PLL samples, rho=0.7074, p<0.0001, Spearman correlation, two-sided). Significantly deregulated genes in both the scRNA sequencing data and the respective bulk sequencing data set are highlighted by a red dot. **(C)** Differential expression of DEGs derived from scRNA sequencing between active-disease PBMCs and healthy-donor T cells was compared between different data sets. Log2 fold-changes derived via scRNA sequencing were correlated to log2 fold-changes from a publicly available gene expression array data set[4] (upper panel, CD3+ T cells of 10 healthy donors vs 69 T-PLL samples, rho=0.7854, p<0.0001, Spearman correlation, two-sided) and a mRNA sequencing data set[5] (lower panel, CD3+ T cells of 6 healthy donors vs 48 T-PLL samples, rho=0.761, p<0.0001, Spearman correlation, two-sided). Genes that were significantly deregulated in both the scRNA sequencing data and the respective bulk sequencing data set are highlighted by a red dot. Despite the tumor purity of utilized T-PLL samples being >95% in the publicly available gene expression data sets, derived differential gene expression from these data correlated more strongly to scRNA sequencing-derived gene expression calculated on PBMCs than to gene expression calculated on T-PLL cells only (B). Source data and complete summaries of statistical analyses are provided in the Source Data file.

Supplemental Figure 2

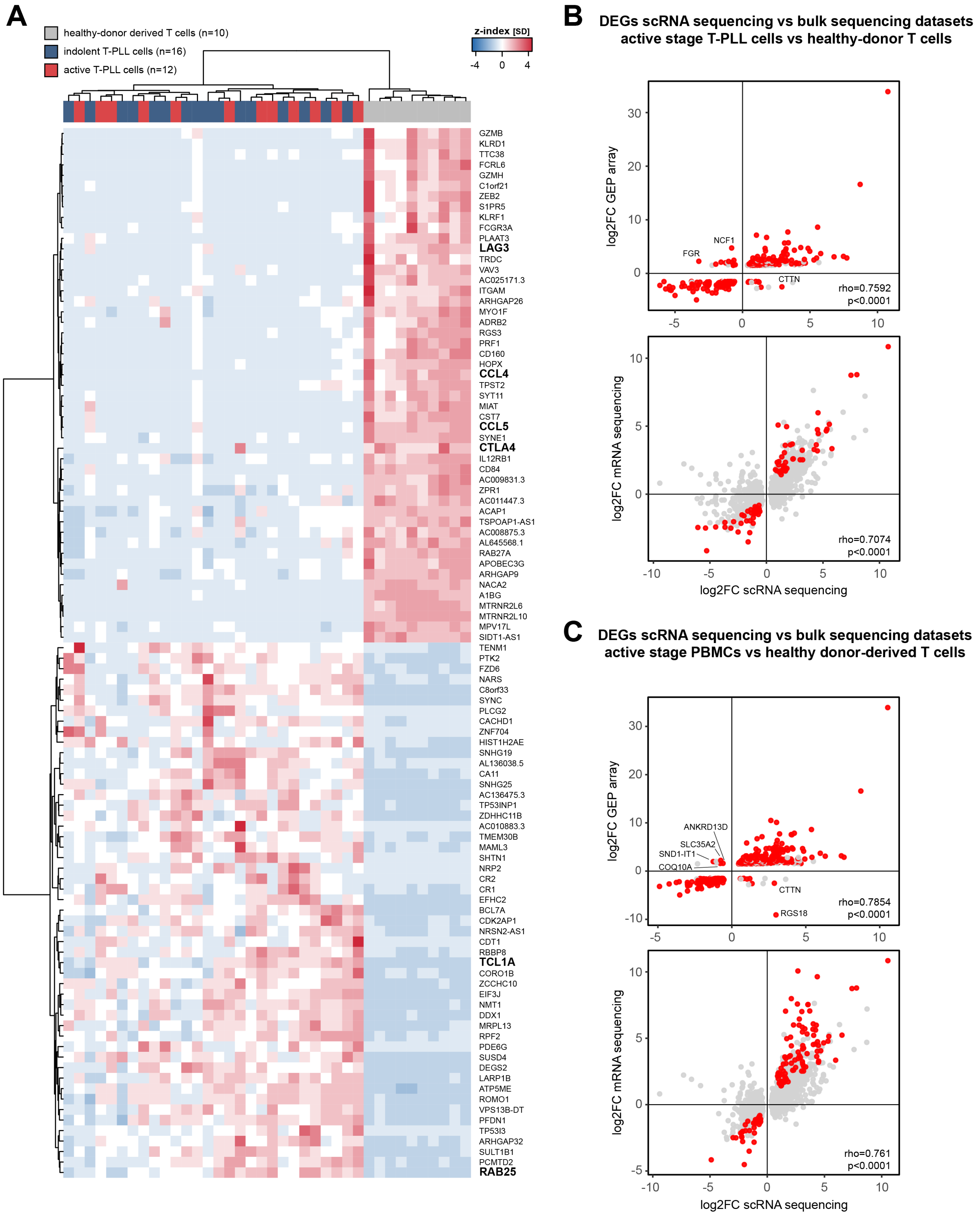

Supplemental Figure 3

(A) Heatmap displaying log2 fold-changes of genes consistently deregulated in at least 4/11 sample pairs. Color indicates the log2 fold-change between active and indolent T-PLL cells (red: upregulated in active T-PLL cells, blue: downregulated in active T-PLL cells). Clustering of patients was based on Spearman distance values. Font face and color of gene labels indicate the number of sample pairs showing deregulation of the respective gene (bold black: deregulated in 8 pairs, regular black: deregulated in 7 pairs, regular grey: deregulated in 4-6 pairs). Genes are functionally grouped based on their KEGG pathway annotation.[6] **(B)** UpSet plot[7] displaying the overlap of significant DEGs among paired T-PLL samples. DEGs were calculated comparing active to indolent T-PLL cells. Source data and complete summaries of statistical analyses are provided in the Source Data file.

Supplemental Figure 3

A Recurrently deregulated genes between indolent and active T-PLL cell samples

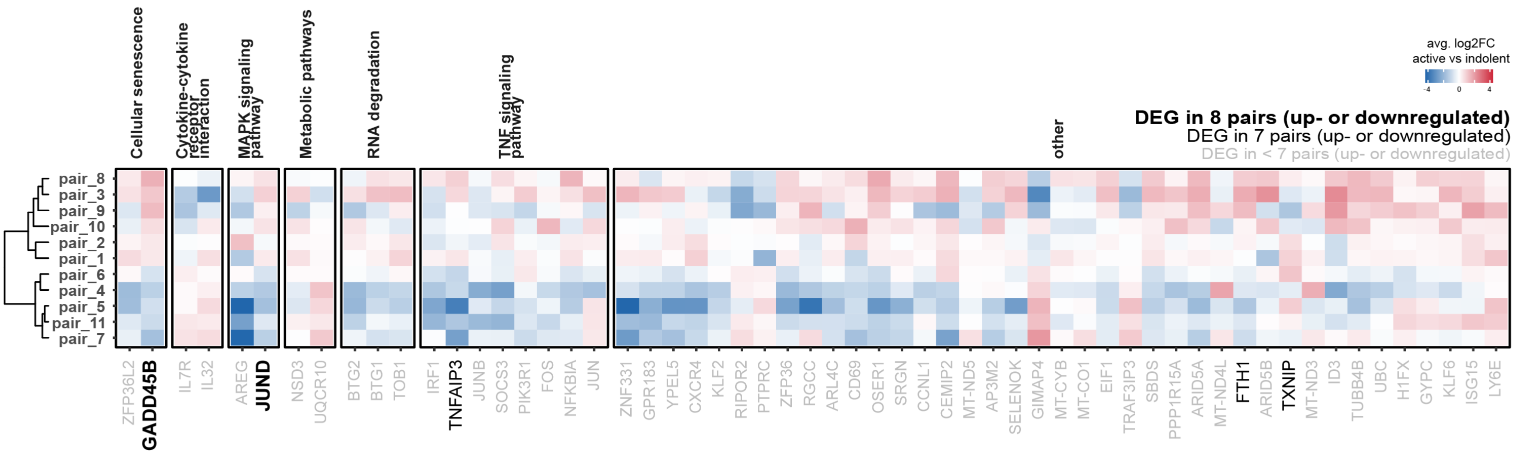

B Overlap of DEGs between active and indolent stage T-PLL cells in paired samples

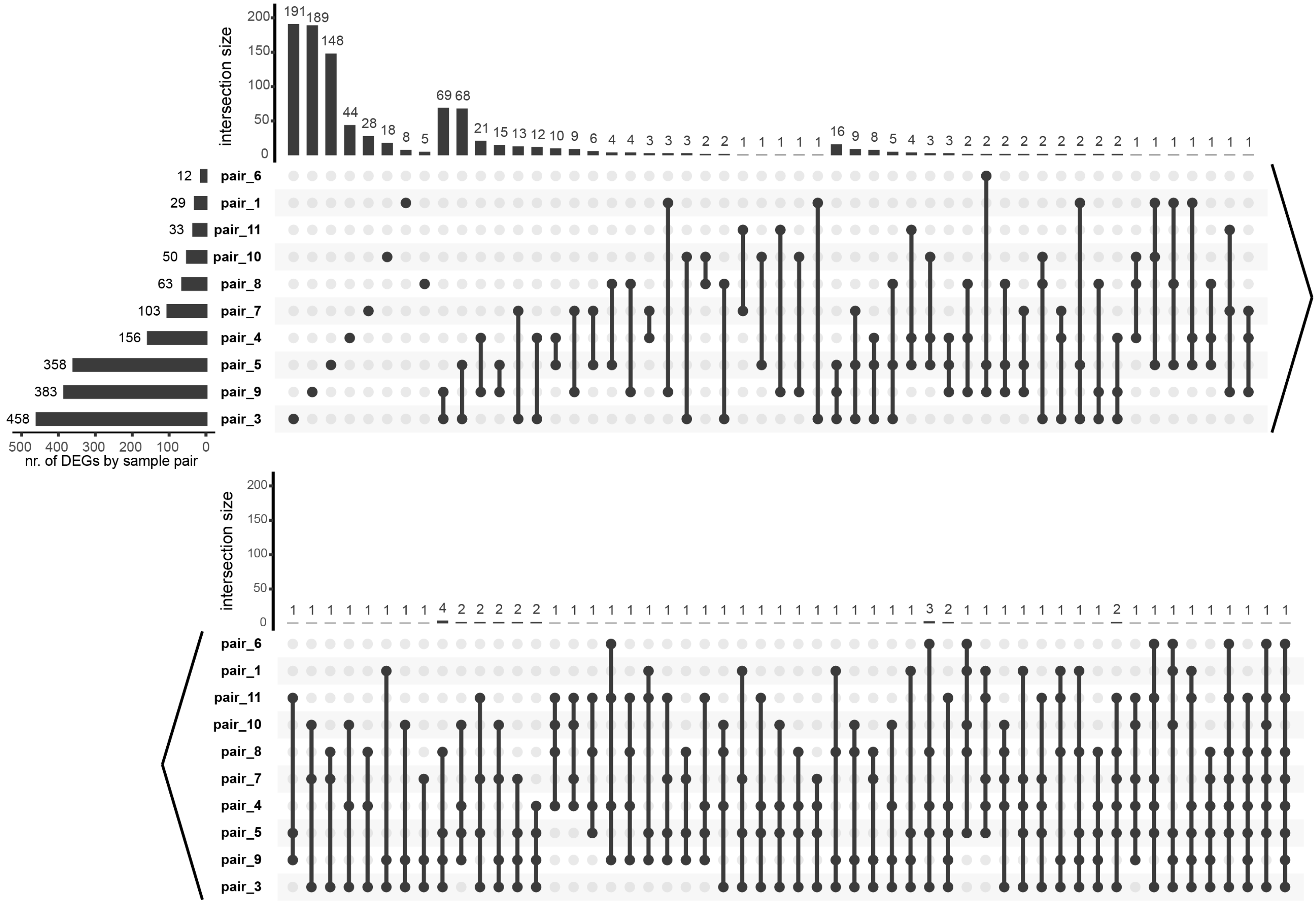

Supplemental Figure 4

(A) Box-whisker-charted mean expression scores of the HALLMARK TNFα/NFκB signaling gene set.[8] Healthy-donor derived T cells[1] were compared to indolent and active-stage T-PLL cells. During the transition to active stage, T-PLL tumor cells displayed a marked reduction in NFκB activity ($p=0.0679$, permutation test, B=10,000, two-sided). **(B)** Mean HALLMARK TNFα/NFκB signaling expression scores of shrinking and expanding T-PLL cell clusters in paired T-PLL samples. Strong downregulation of NFκB activity was observed in expanding clusters of patients pair 4, pair 5, pair 7, and pair 11 that presented a comparably high TNFα/NFκB signaling gene expression signature during indolent disease. Interestingly, some patients (pair 1, pair 3, pair 8) showed an opposite behavior with a strong activation of TNFα/NFκB signaling (****: $p<0.0001$, ***: $p<0.001$, **: $p<0.01$, *: $p<0.05$, MWW, two-sided). **(C)** Color-coded cluster activation scores of the HALLMARK TNFα/NFκB signaling gene set projected on the t-SNE representation of all samples (red: high gene set activity, white: average gene set activity, blue: low gene set activity). Activity of TNFα/NFκB signaling presented a strong influence on the t-SNE representation. **(D)** Box-whisker-charted gene expression of relevant NFκB target genes in T-PLL cells comparing all T-PLL samples. Both *NFKBIA* (top) and *TNFAIP3* (bottom) were upregulated predominantly in indolent T-PLL cells and showed downregulation from indolent to active disease. **(E)** Correlation of gene expression between *NFKBIA* and *GADD45B* in T-PLL cells. GADD45B, a major protein involved in the signal transduction from NFκB to MAPK signaling and a regulator of apoptosis, was associated with *NFKBIA* gene expression (rho=0.288, $p<0.0001$, Spearman correlation, two-sided). **(F)** Box-whisker-charted gene expression of NFκB target genes comparing healthy-donor derived CD3+ T cells (n=6) and active-stage T-PLL samples (n=48) in a publicly available mRNA bulk sequencing[5]. Active T-PLL samples presented comparable levels of gene expression of *NFKBIA* (left), TNFAIP3 (middle), and GADD45B (right) as healthy-donor derived T cells (MWW, two-sided). Definition of box plots: centre: 50th percentile, box bounds: 25th and 75th percentiles (IQR), whiskers: smallest and largest observations (A) within 1.5×IQR of the box (B,D,F). Source data and complete summaries of statistical analyses are provided in the Source Data file.

Supplemental Figure 4

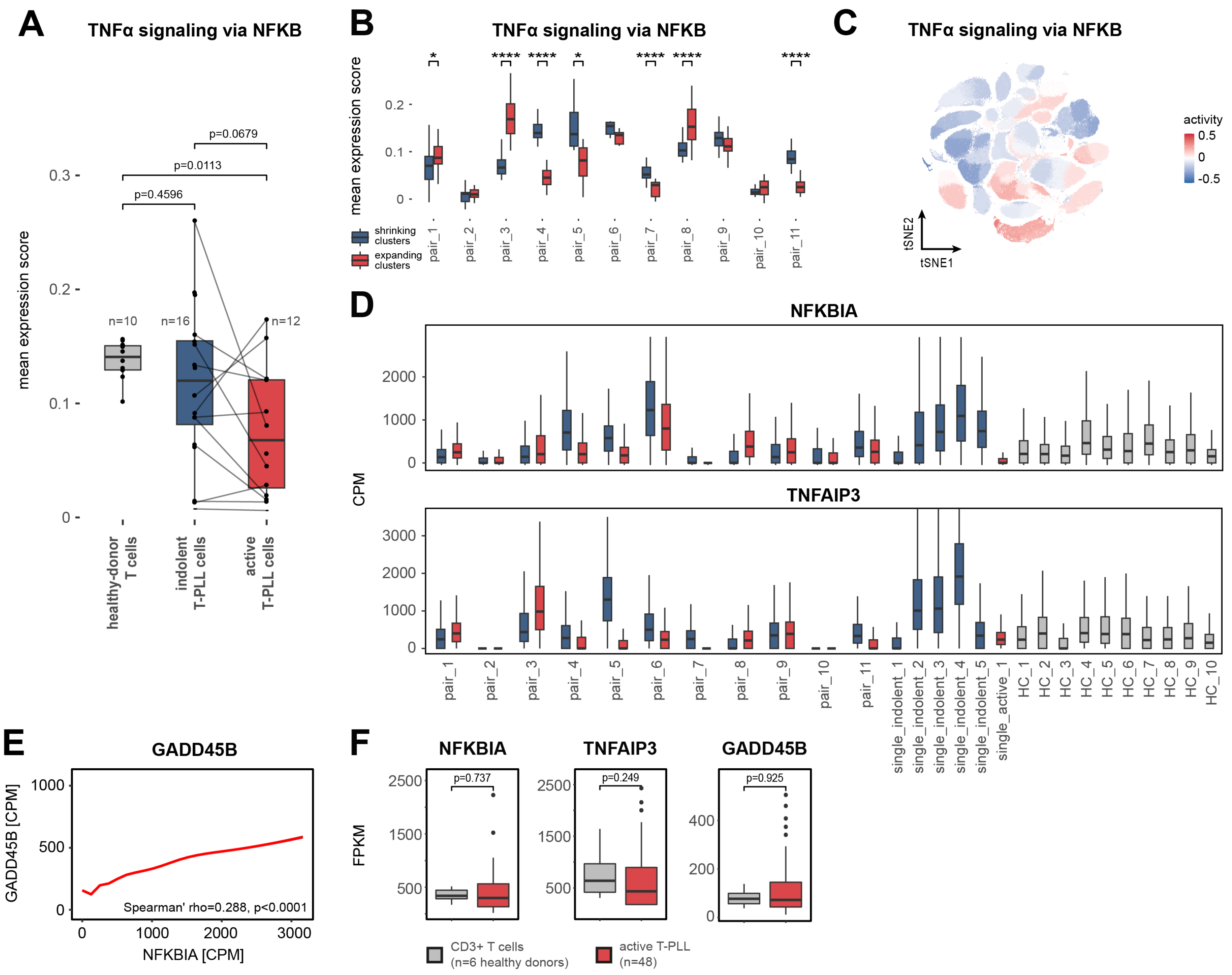

Supplemental Figure 5

(A) GSEA enrichment of most abundant HALLMARK pathways[8] between active and indolent T-PLL cells. Dot colors represent the normalized enrichment score (NES) of pathways (red: enrichment in active T-PLL cells, blue: enrichment in indolent T-PLL cells). FDR is displayed by the size of dots. **(B)** GSEA enrichment of most abundant KEGG gene sets[6] between active and indolent T-PLL cells. Dot colors represent the normalized enrichment score (NES) of pathways (red: enrichment in active T-PLL cells, blue: enrichment in indolent T-PLL cells). FDR is displayed by the size of dots. **(C)** Summarized GSEA enrichment scores of the Reactome pathway collection.[9] Proportion of enriched sub-pathways comparing active to indolent-stage T-PLL cells is encoded by the size of dots for each top-level pathway. Dot colors represent the mean normalized enrichment score (NES) of pathways (red: enrichment in active T-PLL cells, blue: enrichment in indolent T-PLL cells). **(D)** Pairwise t-SNE-representation of integrated T-PLL cells indicating the sample of origin for each cell (top row, blue: indolent T-PLL sample, red: active T-PLL sample) and the assignment to shrinking, stable, and expanding T-PLL cell clusters (bottom row, red: expanding T-PLL cluster, grey: stable T-PLL cluster, blue: shrinking T-PLL cluster). **(E)** Pairwise gene ontology (GO) analysis of DEGs between expanding and shrinking clusters. Bar charts illustrate the enrichment of most strongly enriched terms for upregulated (red) and downregulated (blue) DEGs. **(F)** GSEA enrichment of most abundant KEGG gene sets[6] between expanding and shrinking T-PLL cell clusters. Dot colors represent the normalized enrichment score (NES) of pathways (red: enrichment in expanding T-PLL cell clusters, blue: enrichment in shrinking T-PLL cell clusters). FDR is displayed by the size of dots. **(G)** Summarized GSEA enrichment scores of the Reactome pathway collection.[9] Proportion of enriched sub-pathways comparing expanding to shrinking T-PLL cell clusters encoded by the size of dots for each top-level pathway. Dot colors represent the mean normalized enrichment score (NES) of pathways (red: enrichment in expanding T-PLL cell clusters, blue: enrichment in shrinking T-PLL cell clusters). Source data and complete summaries of statistical analyses are provided in the Source Data file.

Supplemental Figure 5

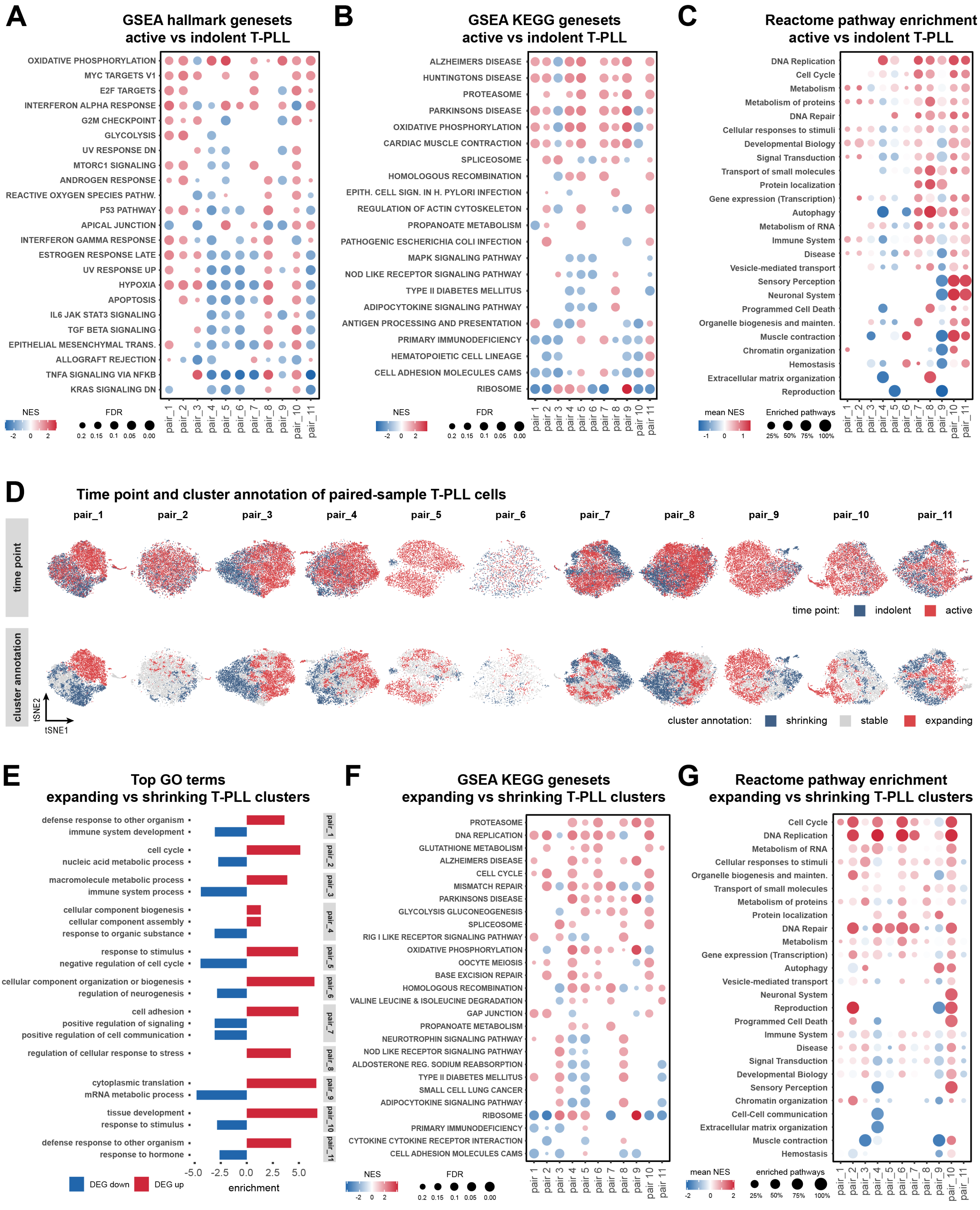

Supplemental Figure 6

(A) Violin plots comparing the distribution of cell-wise derived pseudotime values of T-PLL cells over samples. Linear pseudotime was calculated for each patient using SCORPIUS[10] in Spearman-reduced space. P-values from two-sided MWW tests (****: p<0.0001). Colors indicate the disease stage of samples (blue: indolent, red: active). **(B)** Pseudotime-associated pathway enrichments from GSEA of HALLMARK pathways.[8] Heatmap of NES calculated on pseudotime-associated gene importance values. Colors show the respective NES per pathway and patient (blue: enriched in genes negatively associated with pseudotime progression, red: enriched in genes positively associated to pseudotime progression, white: not significant). Source data and complete summaries of statistical analyses are provided in the Source Data file.

Supplemental Figure 6

A

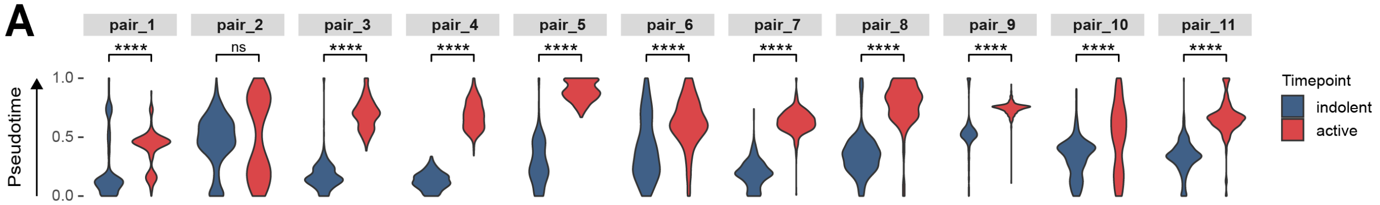

B

GSEA of pseudotime correlated genes

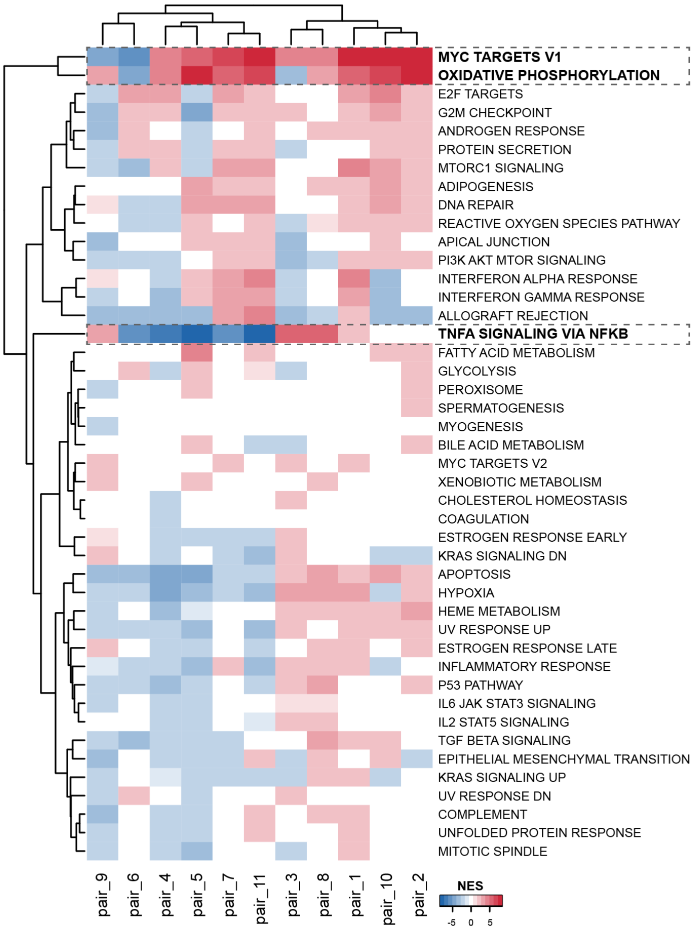

Supplemental Figure 7

(A) Total number and predicted type of functional coding variants in 3 longitudinal sample pairs as derived from WGS. Functional annotation was derived from Ensembl's Variant Effect Predictor release 113.[11] **(B)** Overview on most relevant emerging genomic lesions during disease progression. Dot plots show the allele frequency of variants and predicted copy number of the affected genes. The top 10 functional coding variants with highest delta within their VAF from indolent to active-stage T-PLL are reported for each of the patients (blue dot: indolent stage T-PLL; red dot: active-stage T-PLL). Among the top lesions are variants affecting *ATM* (pair_1), *FASTK* (a strong inducer of lymphocyte apoptosis[12]; pair_2), and *STAT5B* (pair_3). **(C)** Dot plots displaying the temporal dynamics of identified variants within the JAK/STAT signaling pathway. Variant allele frequencies are displayed for the indolent and active time point. An increase in VAF was observed for *JAK3* p.L875H (pair_1) and *STAT5B* p.T628S (pair_3) during progression, whereas *STAT5B* p.N642H (pair_2) and *STAT5B* p.S635T (pair_3) showed a decline. Source data and complete summaries of statistical analyses are provided in the Source Data file.

Supplemental Figure 7

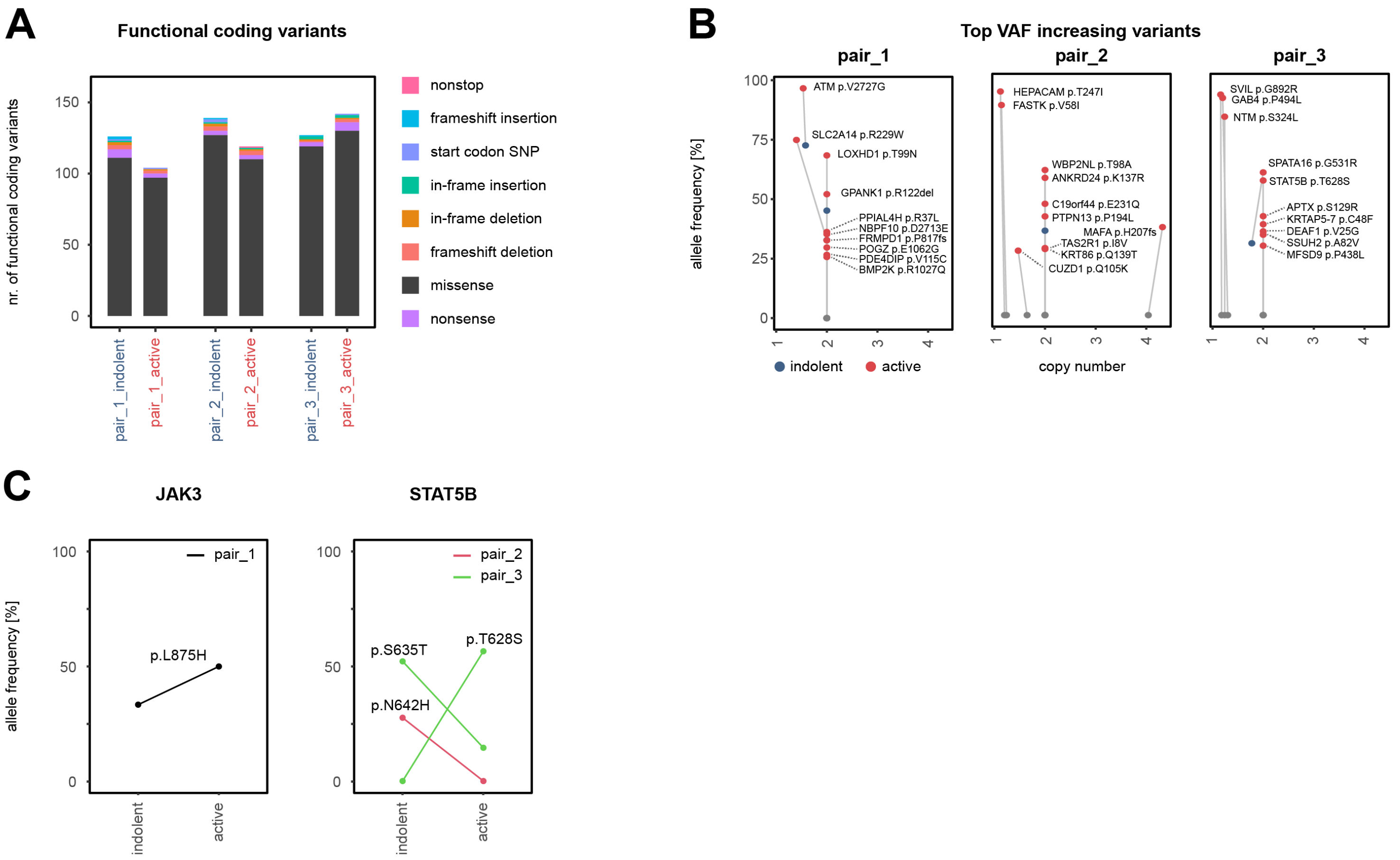

Supplemental Figure 8

(A) Summary of predicted copy number aberrations of T-PLL cells. Copy number states were inferred using InferCNV (v1.9.0, Trinity CTAT Project, Broad Institute of MIT and Harvard) and simplified to the level of chromosome arms. T cells of age-matched healthy donors were used as a control.[1] Tile colors and displayed numbers represent the percentage of T-PLL cells within each sample that harbor the respective copy number change (blue: loss in 100% of T-PLL cells, white: no T-PLL cells with respective copy number alteration, red: gain in 100% of T-PLL cells). **(B)** Differential copy number alterations in paired T-PLL samples. Heatmap displaying the percentage of T-PLL cells that increased (red) or decreased (blue) in their predicted copy number (see A) from the indolent to the active-disease sample. **(C)** Violin plot depicting the percentage of genome affected by copy number alterations in T-PLL cells. Distributions were compared between indolent and active time point-derived T-PLL cells of paired samples using two-sided MWW. CN: copy number. **(D)** Box-whisker plot comparing the mean percentage of copy number-altered genome in T-PLL cells between indolent and active T-PLL samples. There was no significant difference between indolent and active T-PLL (p=0.347, MWW, two-sided). Box plots: centre: 50th percentile, box bounds: 25th and 75th percentiles (IQR), whiskers: smallest and largest observations within 1.5×IQR of the box. Source data and complete summaries of statistical analyses are provided in the Source Data file.

Supplemental Figure 8

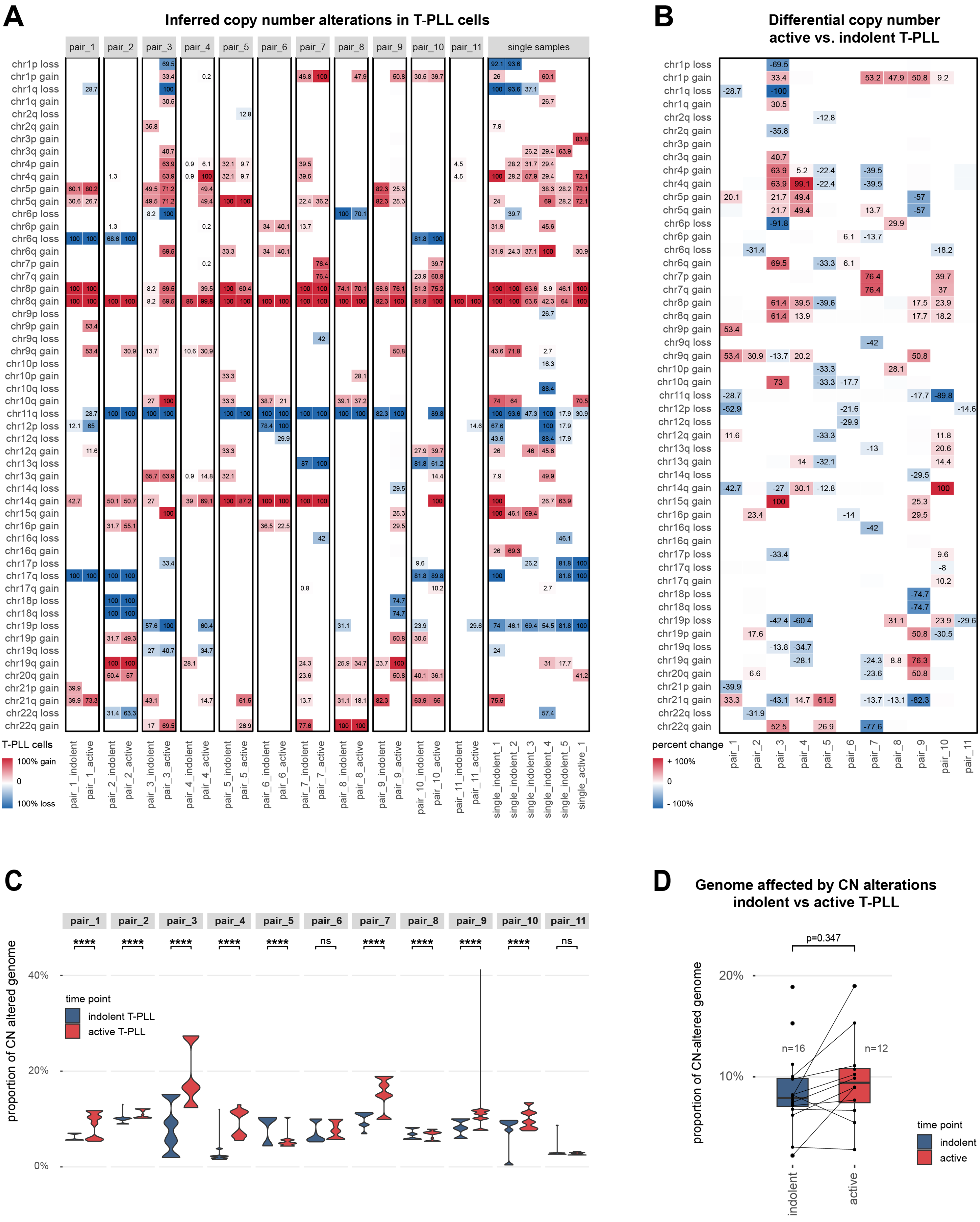

Wahnschaffe L et al., Mechanisms of disease progression in T-PLL

Supplemental Figure 9

(A) High confidence structural variants (SVs) derived from WGS. SVs are displayed for each sequenced sample showing translocations (pink lines), inversions (black lines), as well as copy number alterations (gains: red bars; losses: blue bars). The height of bars represents the inferred copy number, with missing bars implying no copy number alteration at the specific site. The majority of inferred variants are already present at the indolent disease stage. Copy number gains of chromosome 8q, including the locus of *MYC*, are further enriched in all three analyzed T-PLL patients. **(B)** Key structural lesions of sequenced T-PLL samples leading to a juxtaposition of the TCRAD locus and *TCL1A* on chromosome 14. Breakpoints associated with the inv(14)(q11q32) observed in patients pair_1 and pair_3, as well as the t(14;14)(q11;q32) seen in pair_2 (breakpoints extracted manually), are consistent with previously published data.[4] **(C)** Correlation plot comparing derived copy numbers of *MYC* and *ATM* between scRNA expression and WGS-based strategies. Copy numbers were inferred from scRNA data using InferCNV and from WGS data using ClinSV[13] in 6 T-PLL samples. Overall, we observed a high correlation between both methods for T-PLL key genomic lesions (*MYC*: r=0.9816, p=0.0005; *ATM*: r=0.9817, p=0.0005, Pearson correlation). Source data and complete summaries of statistical analyses are provided in the Source Data file.

Supplemental Figure 9

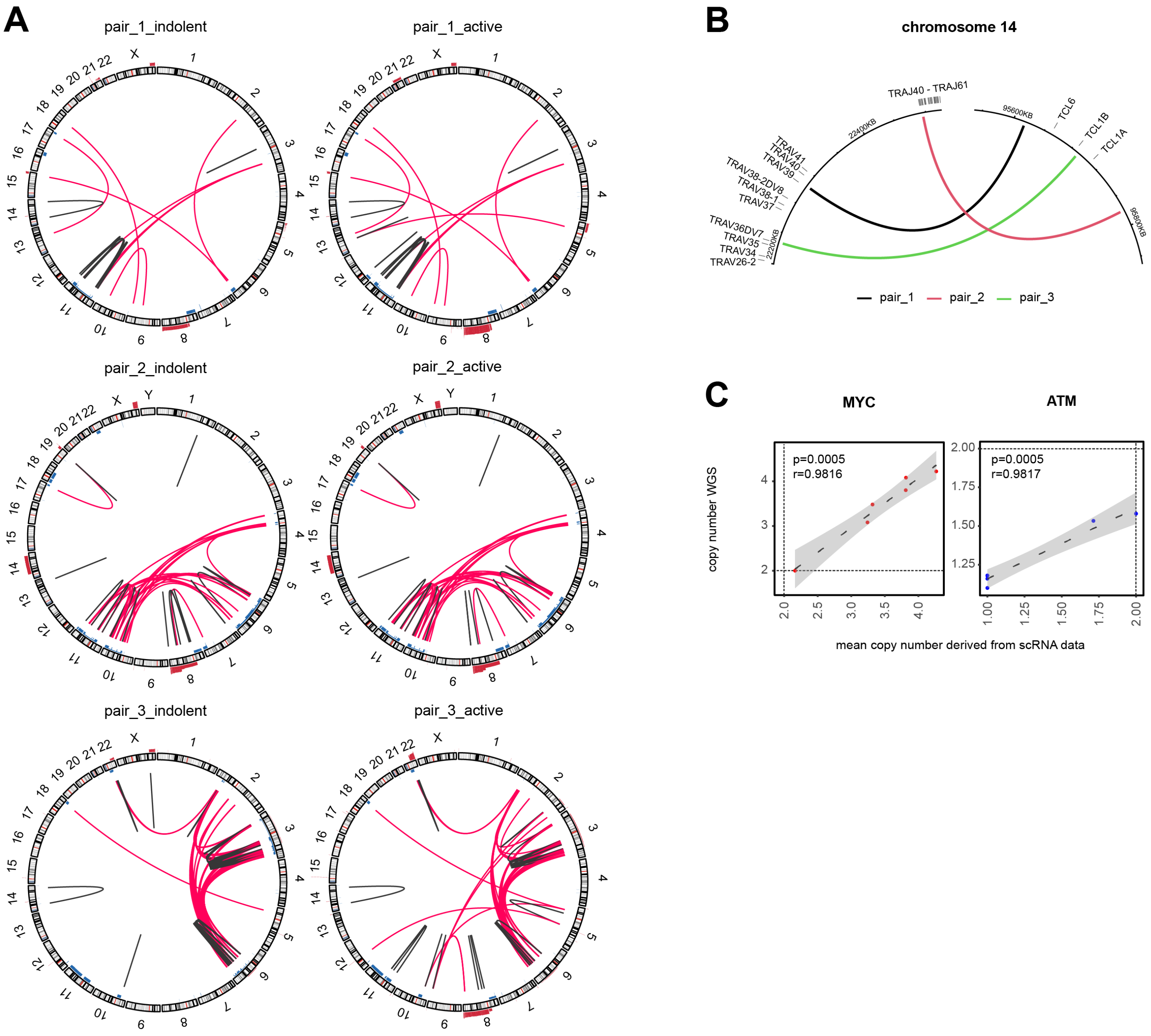

Supplemental Figure 10

(A) Box-whisker charted mean expression score of the HALLMARK MYC targets V2 gene set.[8] Scores were calculated on the merged data set of T-PLL cells and healthy-donor T cells using Seurat's AddModuleScore function.[3] **(B)** Mean expression scores of HALLMARK MYC targets V2 of shrinking and expanding T-PLL cell clusters in paired T-PLL samples (****: p<0.0001, ***: p<0.001, **: p<0.01, *: p<0.05, MWW, two-sided). **(C)** Ridgeline plot comparing cell-based expression levels of *MYC* mRNA in indolent and active-stage T-PLL cells of paired T-PLL samples. Vertical lines indicate log1p mean expression of indolent (blue) and active (red) T-PLL cells. **(D)** Mean inferred copy number of *MYC*. Left: Comparison of indolent and active-stage T-PLL cells of paired T-PLL samples. Active-stage T-PLL cells presented an increase of *MYC* copy number in 7/11 (63.6%) paired T-PLL samples (p=0.118, MWW, two-sided). Right: Comparison of shrinking and expanding T-PLL cell clusters. Expanding T-PLL clusters were associated with a significantly higher *MYC* copy number (p<0.0001, MWW, two-sided). **(E)** Cell-based correlation between inferred *MYC* copy number state and *MYC* expression. Copy number gains of *MYC* were significantly correlated to an increase of *MYC* mRNA (rho=0.268, p<0.0001, Spearman correlation, two-sided). Colors indicate the predicted copy number state: white: 2, pink: 3, light red: 4, dark red: >4. **(F)** Correlation between *MYC* mRNA expression and HALLMARK MYC targets V1 expression scores in n=1000 T-PLL cell pseudo-bulks. There was a significant association between *MYC* mRNA levels and MYC target gene expression (r=0.865, p<0.0001, Pearson correlation, two-sided, grey band: 95% confidence interval). **(G)** Mean HALLMARK MYC targets V1 gene expression scores correlated with mean expression scores of energy metabolism gene sets in shrinking and expanding T-PLL cell clusters of paired T-PLL samples. Upper panel: HALLMARK oxidative phosphorylation (rho=0.748, p<0.0001, Spearman correlation, two-sided). Lower panel: HALLMARK glycolysis (rho=0.724, p<0.0001, Spearman correlation, two-sided). Grey band: 95% confidence interval. Definition of box plots: centre: 50th percentile, box bounds: 25th and 75th percentiles (IQR), whiskers: smallest and largest observations (A) within 1.5×IQR of the box (B,D,E). Source data and complete summaries of statistical analyses are provided in the Source Data file.

Supplemental Figure 10

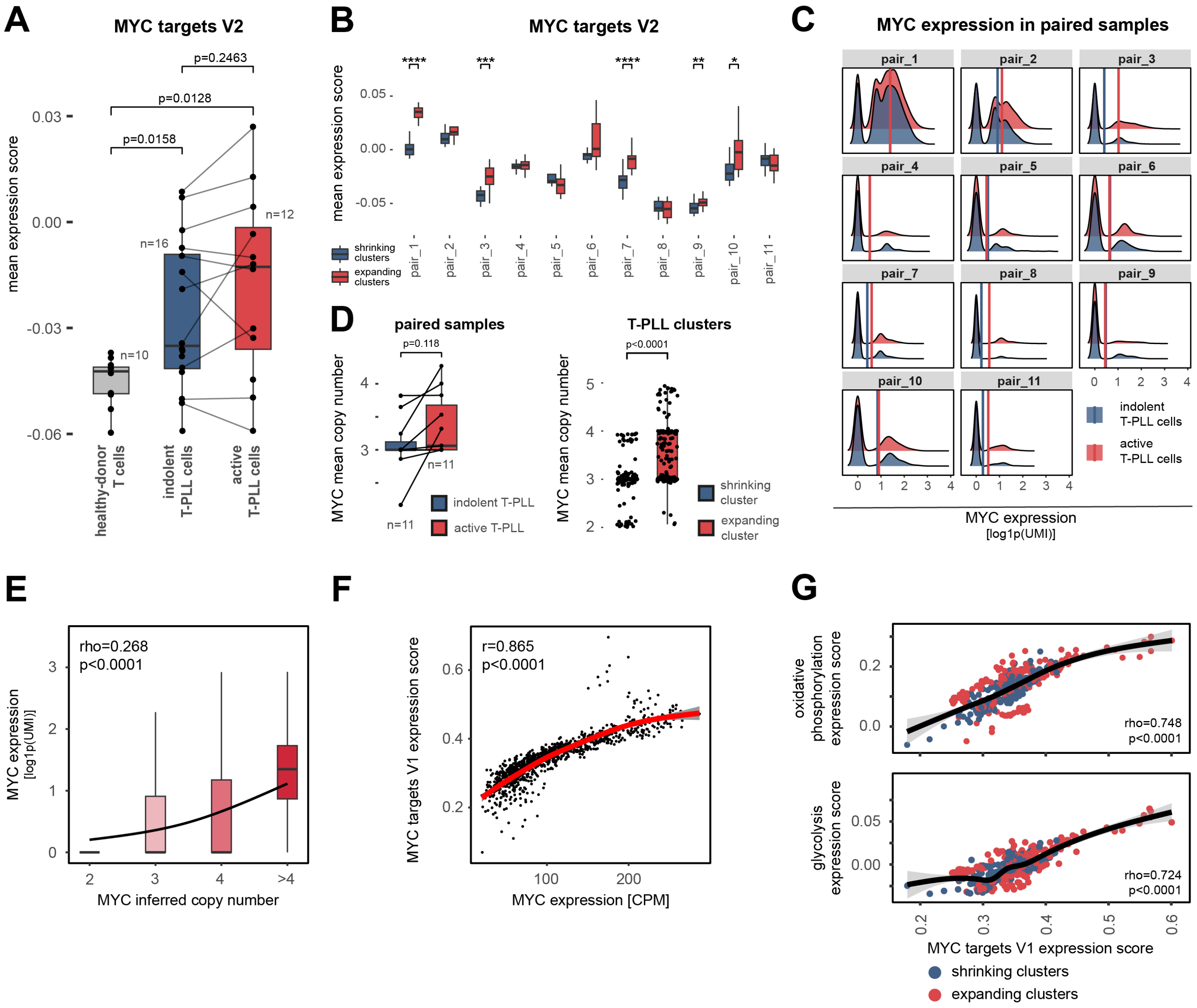

Supplemental Figure 11

(A) Mean HALLMARK oxidative phosphorylation[8] expression scores of shrinking and expanding T-PLL cell clusters in paired T-PLL samples. Expression of oxidative phosphorylation-associated genes was significantly upregulated in expanding T-PLL cell clusters in 7/11 (63.6%) T-PLL patient pairs (****: p<0.0001, ***: p<0.001, **: p<0.01, *: p<0.05, MWW, two-sided). **(B)** Mean HALLMARK glycolysis expression scores of shrinking and expanding T-PLL cell clusters in paired T-PLL samples (****: p<0.0001, ***: p<0.001, **: p<0.01, *: p<0.05, MWW, two-sided). **(C)** Box-whisker plots illustrating the percentage of transcripts encoding for cell metabolism-associated genes in all protein-coding transcripts of T-PLL cells. Indolent-stage T-PLL cells were compared to active T-PLL cells in paired T-PLL samples. The majority of paired samples display a significant upregulation of metabolic transcripts compared to indolent time point T-PLL cells (****: p<0.0001, ns: not significant, MWW, two-sided). **(D)** Correlation of *ex vivo* respiratory capacity and mean HALLMARK oxidative phosphorylation gene expression scores in T-PLL cells. Respiratory capacity was measured using Seahorse XFe 96 assays (Agilent) corroborating a strong association between gene expression of oxidative phosphorylation scores and *ex vivo* respiratory capacity (rho=0.56, p=0.076, Spearman correlation, two-sided). **(E)** Correlation of *ex vivo* glycolytic reserve and mean HALLMARK glycolysis gene expression scores in T-PLL cells. Glycolytic reserve was measured using Seahorse XFe 96 assays (Agilent). Mean gene expression scores of T-PLL cells significantly correlated with the *ex vivo* glycolytic reserve (rho=0.68, p=0.022, Spearman correlation, two-sided). Definition of box plots: centre: 50th percentile, box bounds: 25th and 75th percentiles (IQR), whiskers: smallest and largest observations within 1.5×IQR of the box. Source data and complete summaries of statistical analyses are provided in the Source Data file.

Supplemental Figure 11

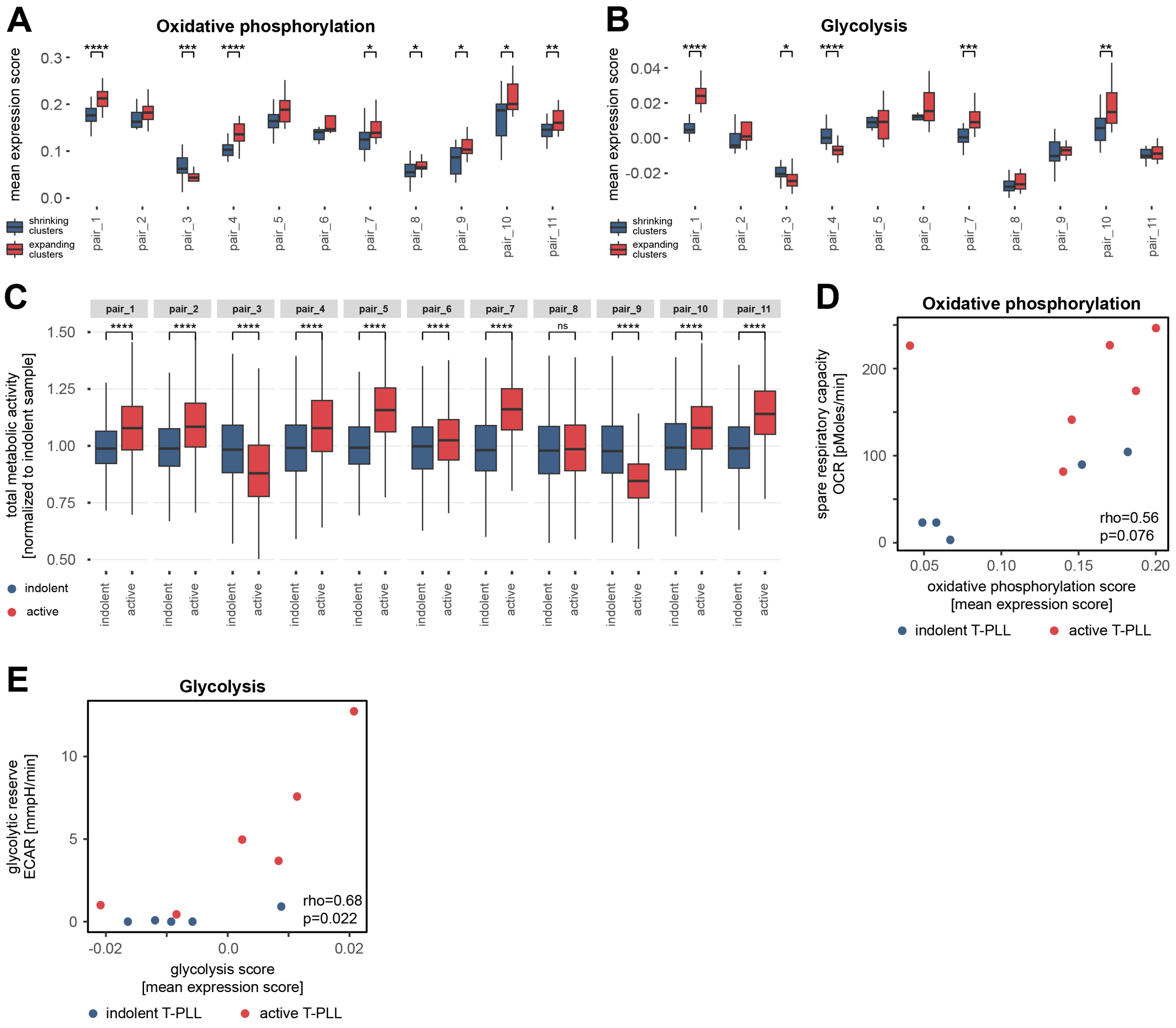

Supplemental Figure 12

Gating strategy used for the analysis of activation marker surface expression by flow cytometry following TCR stimulation via anti-CD3/CD28 crosslinking (corresponding to **Figure 5F**). Expression levels were compared between $CD45^{+}$ and $CD45^{-}$ T-PLL cells from the indolent disease stage of patient pair 1.

Supplemental Figure 12

pair_1 indolent disease stage

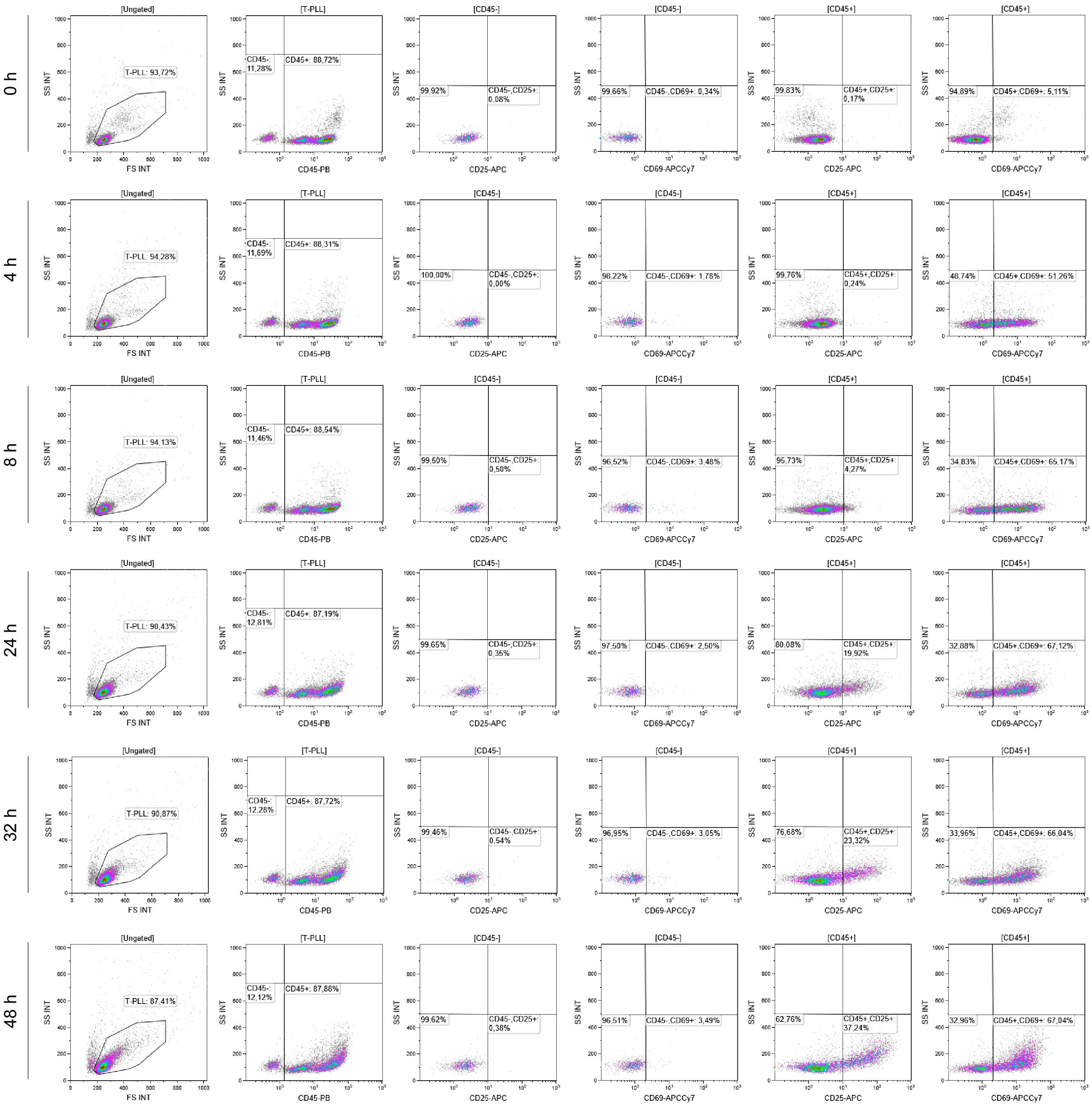

Supplemental Figure 13

Gating strategy used for the analysis of activation marker surface expression by flow cytometry following TCR stimulation via anti-CD3/CD28 crosslinking (corresponding to **Figure 5F**). Expression levels were compared between $CD45^{+}$ and $CD45^{-}$ T-PLL cells from the active disease stage of patient pair 1.

Supplemental Figure 13

pair_1 active disease stage

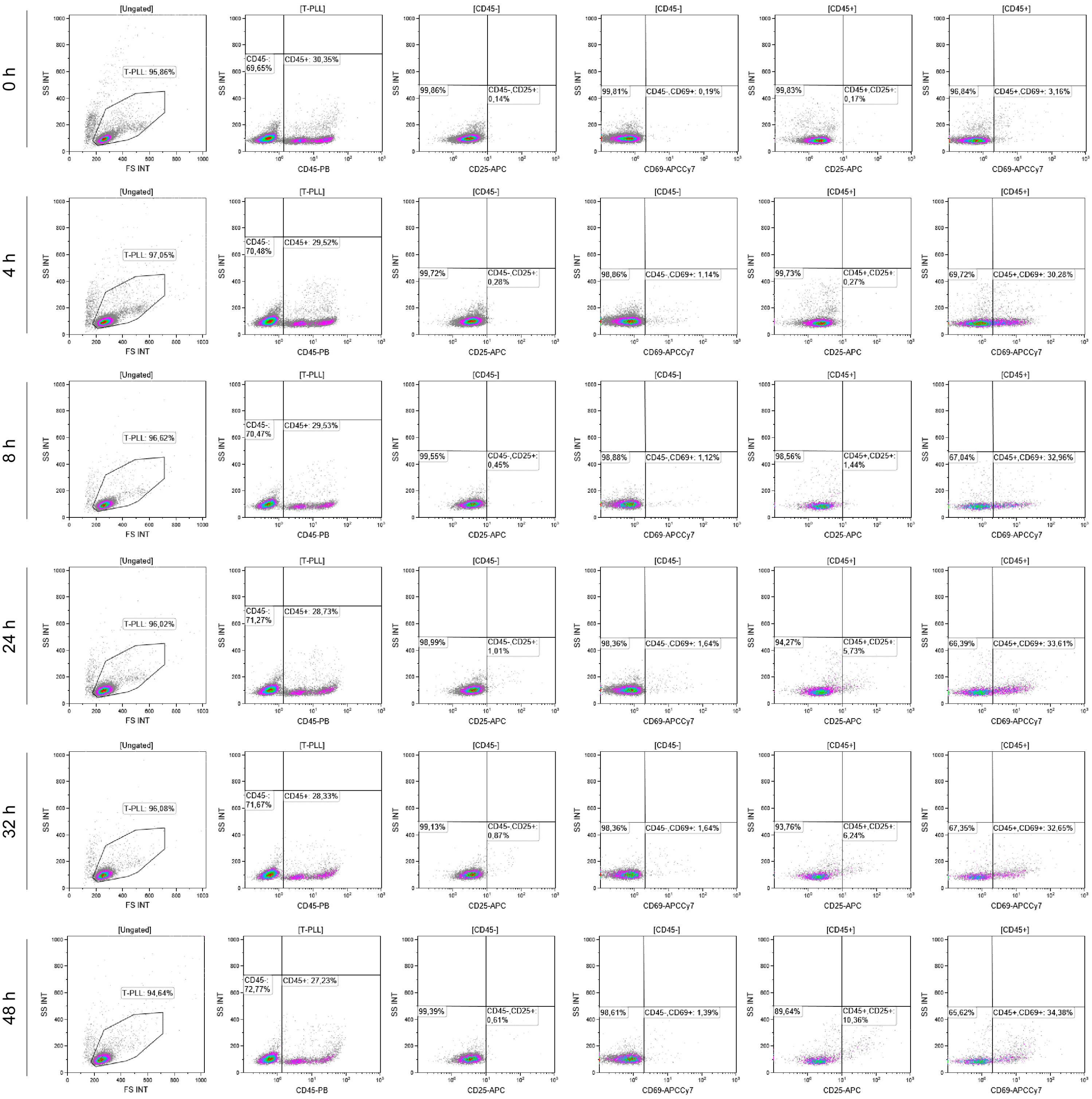

Supplemental Figure 14

(A) Heatmap of the top 25 differentially enriched transcription factor signatures between indolent and active-stage T-PLL cells. Transcription factor enrichment was determined using the DoRothEA gene regulatory network.[14] Enrichment values were scaled, and z-scores were color encoded (red: positive z-score, blue: negative z-score). **(B)** Box-whisker plot displaying the relative contribution of dendritic cells to the non-tumor PBMCs of healthy controls (n=10), as well as indolent (n=16) and active-stage (n=11) T-PLL samples. Dendritic cells are significantly enriched in indolent-disease T-PLL samples (p=0.0002, MWW, two-sided) compared to healthy-donor derived samples[1] and tended to be further increased in active disease (p=0.076, MWW, two-sided). **(C)** Box-whisker plot displaying absolute monocyte counts of indolent (n=9) vs active (n=22) T-PLL patients. Active-stage T-PLL patients presented higher monocyte counts than indolent T-PLL patients (p=0.0005, MWW, two-sided). **(D)** Bar chart showing the relative contribution of CD14 and CD16 monocytes comparing healthy-donor derived PBMCs[1] with PBMCs from indolent and active-stage T-PLL samples. Monocytes were classified as 'CD14 monocytes' or 'CD16 monocytes' by unsupervised projection on an annotated CITE-sequencing data set.[3] Indolent T-PLL PBMCs showed a significantly higher contribution of CD16 monocytes than healthy-donor PBMCs (p<0.0001, chi-squared test) and active T-PLL PBMCs (p<0.0001, chi-squared test). Definition of box plots: centre: 50th percentile, box bounds: 25th and 75th percentiles (IQR), whiskers: smallest and largest observations within 1.5×IQR of the box. Source data and complete summaries of statistical analyses are provided in the Source Data file.

Supplemental Figure 14

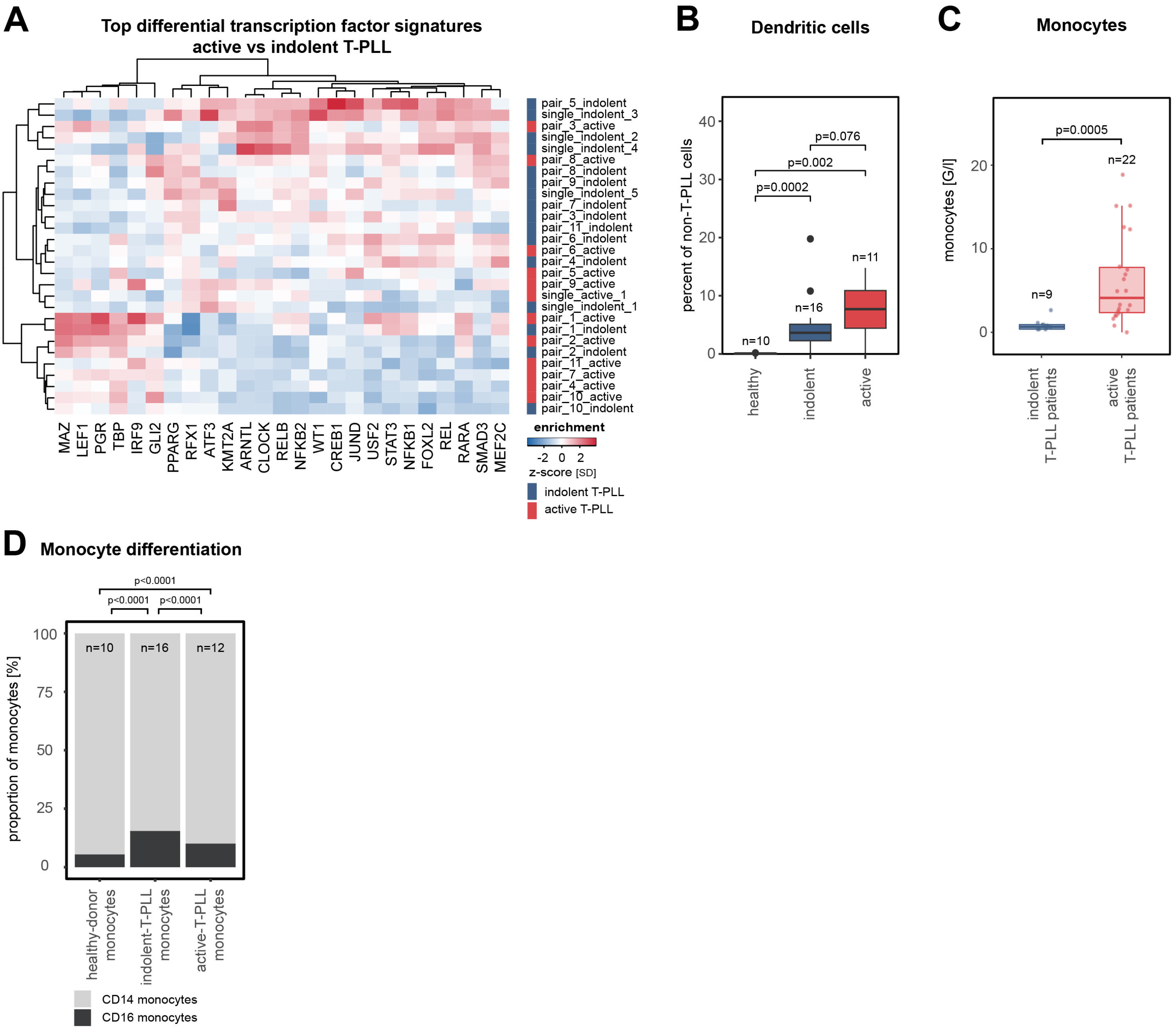

Supplemental Figure 15

(A) Heatmap of most deregulated genes among major non-tumor cell types of active vs indolent T-PLL samples. Differential gene expression was calculated on pseudo-bulks using edgeR LRT[15]. Color indicates the log2 fold-change between active and indolent-stage non-tumor cells (red: upregulated in active T-PLL; white: no change in gene expression; blue: downregulated in active T-PLL). Clustering of patients was based on Euclidean distance values. **(B)** Differential weight of cell-cell interactions between non-tumor PBMCs and indolent vs active-stage T-PLL cells stratified by PBMC cell types and incoming vs outgoing interactions. Colors represent differences in interaction weights between active T-PLL and indolent-stage T-PLL cells (red: increase in the interaction weight of active-stage T-PLL cells, blue: decrease in the interaction weight of active-stage T-PLL cells).[24] **(C)** Overview of top ligands in the deregulation of monocytes from indolent to active-stage T-PLL. Ligands were inferred for each patient separately using NicheNet.[16] Numbers and colors indicate the respective rank in importance for each of the patients (dark red: high rank, white: low rank). **(D)** Top predicted ligands driving monocyte differential gene expression in paired T-PLL samples. Ligands were identified using NicheNet.[16] Colors represent differential gene expression (log2 fold change) in T-PLL cells comparing active and indolent stage (red: upregulated in active T-PLL cells; white: no change in gene expression; blue: downregulated in active T-PLL cells). CD48 was recurrently downregulated in active-stage T-PLL cells. Source data and complete summaries of statistical analyses are provided in the Source Data file.

Supplemental Figure 15

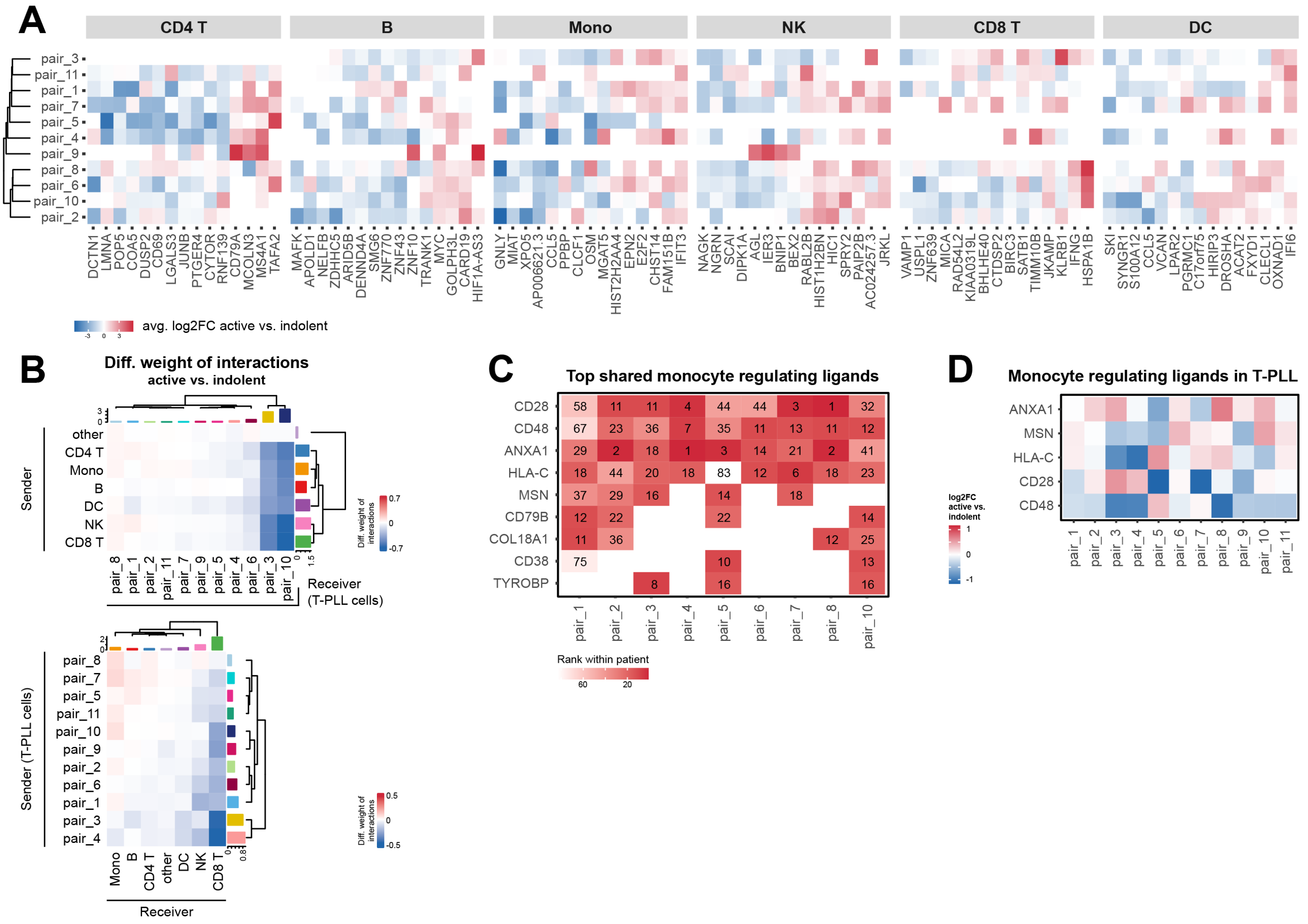

Supplemental Tables

Supplemental Table 1

Overview on sequenced cells:

Sample	Number of sequenced cells	Cells passing quality control		T-PLL cells	
		Number of cells	Relative [%]	Number of cells	Relative [%]
pair_1_indolent	7509	6862	91.38	6281	91.53
pair_1_active	6766	6316	93.35	6128	97.02
pair_2_indolent	4901	4420	90.19	3941	89.16
pair_2_active	5048	4735	93.8	4452	94.02
pair_3_indolent	7237	6704	92.64	6259	93.36
pair_3_active	7941	7187	90.5	6654	92.58
pair_4_indolent	7812	6016	77.01	5456	90.69
pair_4_active	10963	7862	71.71	7363	93.65
pair_5_indolent	550	343	62.36	283	82.51
pair_5_active	6278	4774	76.04	4619	96.75
pair_6_indolent	2800	2063	73.68	1186	57.49
pair_6_active	3044	2222	73	774	34.83
pair_7_indolent	8797	7915	89.97	7118	89.93
pair_7_active	7612	6731	88.43	6443	95.72
pair_8_indolent	16464	11637	70.68	8716	74.9
pair_8_active	12422	9600	77.28	8939	93.11
pair_9_indolent	12504	7025	56.18	1936	27.56
pair_9_active	7535	7362	97.7	7346	99.78
pair_10_indolent	5906	5096	86.29	1655	32.48
pair_10_active	6347	5830	91.85	5437	93.26
pair_11_indolent	6004	5600	93.27	4584	81.86
pair_11_active	4336	4176	96.31	3921	93.89
single_indolent_1	5609	5144	91.71	4950	96.23
single_indolent_2	11549	6341	54.91	5619	88.61
single_indolent_3	13101	12455	95.07	11712	94.03
single_indolent_4	5882	1390	23.63	1309	94.17
single_indolent_5	5561	4828	86.82	3902	80.82
single_active_1	11808	9481	80.29	8999	94.92

Supplemental Table 2:

Primers:

Primer	Sequence 5'-3'
IL32_for	TGGCGGCTTATTATGAGGAGC
IL32_rev	CTCGGCACCGTAATCCATCTC
S100A4_for	GATGAGCAACTTGGACAGCAA
S100A4_rev	CTGGGCTGCTTATCTGGGAAG
CD52_for	TCTTCCTCCTACTCACCATCAG
CD52_rev	CCTCCGCTTATGTTGCTGGA
EZR_for	ACCAATCAATGTCCGAGTTACC
EZR_rev	GCCGATAGTCTTTACCACCTGA
DDX21_for	GAGGAGCCATCTCAAAATGACA
DDX21_rev	GGGTTACAGTCCGGTTCAGG
FTH1_for	CCCCCATTTGTGTGACTTCAT
FTH1_rev	GCCCGAGGCTTAGCTTTCATT
PTPRC_for	ACCACAAGTTTACTAACGCAAGT
PTPRC_rev	TTTGAGGGGGATTCCAGGTAAT
IL7R_for	CCCTCGTGGAGGTAAAGTGC
IL7R_rev	CCTTCCCGATAGACGACACTC
beta-actin_for	TCCCTCACAGCACTAGTATTTCATG
beta-actin_rev	GAATCGGCTGTGTTCTCACAAG

for: forward primer, rev: reverse primer

Supplemental Table 3:

Antibodies:

Application	Antibody	Working conc.	Cat. no.	Manufacturer
FC	anti-CD25 APC (clone BC96)	1:200	302610	BioLegend
FC	anti-CD69 APC/Cy7 (clone FN50)	1:200	310914	BioLegend
FC	anti-CD45 PB (clone HI30)	1:200	304038	BioLegend
WB	anti-β-actin (clone C4)	1:1000	sc-47778	Santa Cruz
WB	anti-c-Myc	1:1000	9402	Cell Signaling
WB	anti-IκBα	1:1000	9242	Cell Signaling
WB	anti-MAPK (Erk1/2) (clone 3A7)	1:1000	9107	Cell Signaling
WB	anti-phospho-MAPK (Erk1/2) (T202/Y204)	1:1000	9101	Cell Signaling
WB	anti-phospho-PLCγ1 (Y783)	1:1000	2821	Cell Signaling
WB	anti-phospho-Zap-70 (Y319) (clone 65E4)	1:1000	2717	Cell Signaling
WB	anti-PLCγ1 (clone D9H10)	1:1000	5690	Cell Signaling
WB	anti-Zap-70 (clone D1C10E)	1:1000	3165	Cell Signaling
WB	polyclonal anti-mouse IgG	1:5000	715-036-150	Jackson ImmunoResearch
WB	polyclonal anti-rabbit IgG	1:5000	711-035-152	Jackson ImmunoResearch

FC: Flow cytometry. WB: Western blot

References

1. Vu, L. T. *et al.* Single-cell transcriptomics of the immune system in ME/CFS at baseline and following symptom provocation. *Cell Rep. Med.* **5**, (2024).
2. Nowotschin, S. *et al.* The emergent landscape of the mouse gut endoderm at single-cell resolution. *Nature 2019 569:7756* **569**, 361–367 (2019).
3. Hao, Y. *et al.* Integrated analysis of multimodal single-cell data. *Cell* **184**, 3573-3587.e29 (2021).
4. Schrader, A. *et al.* Actionable perturbations of damage responses by TCL1/ATM and epigenetic lesions form the basis of T-PLL. *Nat. Commun.* **9**, 697 (2018).
5. Braun, T. *et al.* Micro-RNA networks in T-cell prolymphocytic leukemia reflect T-cell activation and shape DNA damage response and survival pathways. *Haematologica* **107**, 187–200 (2022).
6. Kanehisa, M. Toward understanding the origin and evolution of cellular organisms. *Protein Sci.* **28**, 1947–1951 (2019).
7. Conway, J. R., Lex, A. & Gehlenborg, N. UpSetR: an R package for the visualization of intersecting sets and their properties. *Bioinformatics* **33**, 2938–2940 (2017).
8. Liberzon, A. *et al.* The Molecular Signatures Database (MSigDB) hallmark gene set collection. *Cell Syst.* **1**, 417 (2015).
9. Jassal, B. *et al.* The reactome pathway knowledgebase. *Nucleic Acids Res.* **48**, D498–D503 (2020).
10. Cannoodt, R. *et al.* SCORPIUS improves trajectory inference and identifies novel modules in dendritic cell development. *bioRxiv* 079509 (2016) doi:10.1101/079509.
11. Harrison, P. W. *et al.* Ensembl 2024. *Nucleic Acids Res.* **52**, D891–D899 (2024).
12. Tian, Q., Taupin, J. L., Elledge, S., Robertson, M. & Anderson, P. Fas-activated serine/threonine kinase (FAST) phosphorylates TIA-1 during Fas-mediated apoptosis. *J. Exp. Med.* **182**, 865 (1995).
13. Minoche, A. E. *et al.* ClinSV: clinical grade structural and copy number variant detection from whole genome sequencing data. *Genome Med.* **13**, (2021).
14. Garcia-Alonso, L., Holland, C. H., Ibrahim, M. M., Turei, D. & Saez-Rodriguez, J. Benchmark and integration of resources for the estimation of human transcription factor activities. *Genome Res.* **29**, 1363–1375 (2019).
15. Chen, Y. *et al.* From reads to genes to pathways: differential expression analysis of RNA-Seq experiments using Rsubread and the edgeR quasi-likelihood pipeline. *F1000Research 2016 5:1438* **5**, 1438 (2016).
16. Browaeys, R., Saelens, W. & Saeys, Y. NicheNet: modeling intercellular communication by linking ligands to target genes. *Nat. Methods* **17**, 159–162 (2020).
