# Supplementary material for: Single-cell genomics highlight MYC-associated metabolic activation and altered cell interactions in T-prolymphocytic leukemia progression
Source: Nat Commun. 2026 Mar 9;17:2319. doi: 10.1038/s41467-026-70185-w (PMC12976252; doi:10.1038/s41467-026-70185-w)
Supplement: Supplementary file 3 — Description of Additional Supplementary Files [file 41467_2026_70185_MOESM3_ESM.pdf]

Description of Additional Supplementary Files

Supplementary Data 1:

Descriptive overview of analyzed T-PLL cases and sample characteristics.

Supplementary Data 2:

Filtered functional short variants identified by whole genome sequencing in 6 T-PLL samples from 3 patients. Details on data acquisition and processing are provided in the Methods.
