# Supplementary material for: Single-cell genomics highlight MYC-associated metabolic activation and altered cell interactions in T-prolymphocytic leukemia progression
Source: Nat Commun. 2026 Mar 9;17:2319. doi: 10.1038/s41467-026-70185-w (PMC12976252; doi:10.1038/s41467-026-70185-w)
Supplement: Supplementary file 6 — Reporting Summary [file 41467_2026_70185_MOESM6_ESM.pdf]

nature portfolio

Corresponding author(s): Marco Herling

Last updated by author(s): Feb 11, 2026

Reporting Summary

Nature Portfolio wishes to improve the reproducibility of the work that we publish. This form provides structure for consistency and transparency in reporting. For further information on Nature Portfolio policies, see our Editorial Policies and the Editorial Policy Checklist.

Statistics

For all statistical analyses, confirm that the following items are present in the figure legend, table legend, main text, or Methods section.

n/a	Confirmed	
☐	☒	The exact sample size (*n*) for each experimental group/condition, given as a discrete number and unit of measurement
☒	☐	A statement on whether measurements were taken from distinct samples or whether the same sample was measured repeatedly
☐	☒	The statistical test(s) used AND whether they are one- or two-sided<br>*Only common tests should be described solely by name; describe more complex techniques in the Methods section.*
☐	☒	A description of all covariates tested
☐	☒	A description of any assumptions or corrections, such as tests of normality and adjustment for multiple comparisons
☐	☒	A full description of the statistical parameters including central tendency (e.g. means) or other basic estimates (e.g. regression coefficient) AND variation (e.g. standard deviation) or associated estimates of uncertainty (e.g. confidence intervals)
☐	☒	For null hypothesis testing, the test statistic (e.g. *F*, *t*, *r*) with confidence intervals, effect sizes, degrees of freedom and *P* value noted<br>*Give P values as exact values whenever suitable.*
☒	☐	For Bayesian analysis, information on the choice of priors and Markov chain Monte Carlo settings
☒	☐	For hierarchical and complex designs, identification of the appropriate level for tests and full reporting of outcomes
☐	☒	Estimates of effect sizes (e.g. Cohen's *d*, Pearson's *r*), indicating how they were calculated

Our web collection on statistics for biologists contains articles on many of the points above.

Software and code

Policy information about availability of computer code

Data collection	Raw reads were aligned to the GRCh38-2020-A reference genome, filtered, and counted using the 10x Genomics Cell Ranger 6.1.2 pipeline at default parameters. UMI count matrices were then further processed using the R package Seurat v4.0.52 and R version v4.1 (R Foundation for Statistical Computing).
Data analysis	We made use of the following software packages:<br>10x Genomics Cell Ranger 6.1.2<br><br>GSEA v4.1.0 for Linux<br>R version v4.1 (R Foundation for Statistical Computing)<br>R package Seurat v4.0.5<br>R package STACAS v1.1.0<br>R package UpSetR v1.4.0<br>R package scProportionTest v0.0.0.9<br>R package Libra v1.0.0<br>R package SCORPIUS v1.0.8<br>R package topGO v2.44.0<br>R package inferCNV v1.9.0<br>R package decoupleR v2.2.0<br>R package CellChat v2.1.2<br>R package NicheNet v2.2.0<br>R package biomaRt 2.58.2

R package circlize 0.4.16
R package clusterProfiler 4.10.1
R package collapse 2.1.3
R package ComplexHeatmap 2.18.0
R package corrplot 0.95
R package dorothea 1.14.1
R package edgeR 4.0.16
R package future 1.67.0
R package future.apply 1.20.0
R package ggpubr 0.6.1
R package ggrepel 0.9.6
R package ggridges 0.5.7
R package ggsignif 0.6.4
R package ggtext 0.1.2
R package gplots 3.2.0
R package gridExtra 2.3
R package harmony 1.2.3
R package httr 1.4.7
R package KEGGREST 1.42.0
R package Matrix 1.6-5
R package org.Hs.eg.db 3.18.0
R package pals 1.1
R package patchwork 1.3.2
R package pathview 1.42.0
R package pheatmap 1.0.13
R package qvalue 2.34.0
R package RColorBrewer 1.1-3
R package readxl 1.4.5
R package rstatix 0.7.2
R package scales 1.4.0
R package SeuratDisk 0.0.0.9020
R package svglite 2.2.1
R package tidytext 0.4.3
R package tidyverse 2.0.0
R package tools 4.3.3
R package viper 1.36.0

WGS data analysis was performed using Genome Analysis Toolkit 4 (GATK4, Broad Institute) best practices for data preprocessing and somatic short variant discovery in tumor-only samples. All tools were run with default parameters using the GATK V4.5.0.0 Docker container. Reference files were obtained from the GATK hg38 resource bundle (v0, Broad Institute).

For manuscripts utilizing custom algorithms or software that are central to the research but not yet described in published literature, software must be made available to editors and reviewers. We strongly encourage code deposition in a community repository (e.g. GitHub). See the Nature Portfolio guidelines for submitting code & software for further information.

Data

Policy information about availability of data

All manuscripts must include a data availability statement. This statement should provide the following information, where applicable:

- Accession codes, unique identifiers, or web links for publicly available datasets
- A description of any restrictions on data availability
- For clinical datasets or third party data, please ensure that the statement adheres to our policy

Filtered feature barcode matrices and raw scRNA data of all T-PLL samples have been deposited at GEO under accession number GSE238130 [https://www.ncbi.nlm.nih.gov/geo/query/acc.cgi?acc=GSE238130].
Data from 10 age- and sex-matched healthy controls (Vu et al., 2024) were downloaded from GEO GSE214284 [https://www.ncbi.nlm.nih.gov/geo/query/acc.cgi?acc=GSE214284].
Bulk gene expression array data (Schrader et al., 2018) were downloaded from GEO GSE107513 [https://www.ncbi.nlm.nih.gov/geo/query/acc.cgi?acc=GSE107513].
Bulk mRNA sequencing data from Braun et al., 2021 are available at GEO under GSE318878 [https://www.ncbi.nlm.nih.gov/geo/query/acc.cgi?acc=GSE318878].
The whole-genome sequencing (WGS) data generated in this study contain potentially identifiable germline information from patients and are, therefore, subject to data-protection regulations and restrictions mandated by the informed consent and local ethics approval. Access to de-identified raw WGS data is available under restricted access for non-commercial research purposes to qualified researchers whose proposed use is compatible with the original ethics approval and patient consent, and who have obtained approval from their institutional review board or an equivalent ethics committee. Requests for access to WGS data should be directed to the corresponding author (M. Herling, marco.herling@medizin.uni-leipzig.de). Applicants will be asked to provide a short proposal describing the intended use of the data and to sign a data-transfer agreement. Requests will normally be answered within 4 weeks. If access is granted, data will be shared via a secure file-transfer system and will remain available to approved requestors for at least 10 years after publication.
Aggregated and processed data from whole-genome sequencing are provided in supplementary Data 2 and in the Source Data file.
Source data are provided with this paper.

Research involving human participants, their data, or biological material

Policy information about studies with human participants or human data. See also policy information about sex, gender (identity/presentation), and sexual orientation and race, ethnicity and racism.

Reporting on sex and gender	We did not report on gender. Patient's sex was reported as stated by the patients themselves. We designed the study to maintain a comparable sex-distribution between healthy donors and T-PLL patients. We did not perform analyses stratified by sex because the study was not powered at the sample level to support meaningful sex-based comparisons. Sex is provided as metadata and can be considered in future studies.
Reporting on race, ethnicity, or other socially relevant groupings	We did not collect or report data on race, ethicity, or other socially relevant groupings.
Population characteristics	The unpublished core population of this study comprises primary cells of 17 untreated T-prolymphocytic leukemia (T-PLL) patients from six centers. Samples were obtained at different time points during the transition from indolent to active disease stage. Eleven longitudinal sample pairs from T-PLL patients were acquired. The diagnosis of T-PLL was established according to the WHO criteria and T-PLL consensus guidelines. Written informed consent was provided by all patients according to the declaration of Helsinki.
Recruitment	Patients were recruited accross six centers. We included all patients that presented at a primarily indolent T-PLL stage as defined by consensus criteria (Staber PB, Herling M, Bellido M, et al. Consensus criteria for diagnosis, staging, and treatment response assessment of T-cell prolymphocytic leukemia. Blood. 2019;134(14):1132–1143) with available primary material.
Ethics oversight	All patients provided written informed consent and the study was approved by the ethics committees of the University Hospital Cologne (#11–319) and the Medical University of Vienna (1957/2020).

Note that full information on the approval of the study protocol must also be provided in the manuscript.

Field-specific reporting

Please select the one below that is the best fit for your research. If you are not sure, read the appropriate sections before making your selection.

☒ Life sciences ☐ Behavioural & social sciences ☐ Ecological, evolutionary & environmental sciences

For a reference copy of the document with all sections, see nature.com/documents/nr-reporting-summary-flat.pdf

Life sciences study design

All studies must disclose on these points even when the disclosure is negative.

Sample size	Limited availability dictated sample size (orphan disease).
Data exclusions	Following best practices we filtered out poor-quality cells keeping cells with at least 250 and not more than 4500 detected features and less than 20% mitochondrial genes. In total, we retained 204,959 single cells (78.6%, supplemental Table 2).
Replication	We performed successfull technical validation of single DEGs using qRT-PCR (Pearson’s r=0.95, p=0.0003, supplemental Figure 1A; supplemental Table 3).<br><br>Gene expression data was further validated by correlation of identified DEGs to public available bulk gene expression datasets in T-PLL (supplemental Figures 2A-C).<br><br>Further, we repeated all analyses using the supplied analysis code and confirm reproducibility.
Randomization	Samples were allocated to experimental groups based on their clinical disease stage as defined by consensus criteria (Staber PB, Herling M, Bellido M, et al. Consensus criteria for diagnosis, staging, and treatment response assessment of T-cell prolymphocytic leukemia. Blood. 2019;134(14):1132–1143).
Blinding	This was a primarily bioinformatic study based on sequencing data. Investigators were not blinded to sample group allocation during analysis, as group labels were required to define the statistical models.

Reporting for specific materials, systems and methods

We require information from authors about some types of materials, experimental systems and methods used in many studies. Here, indicate whether each material, system or method listed is relevant to your study. If you are not sure if a list item applies to your research, read the appropriate section before selecting a response.

Materials & experimental systems

n/a	Involved in the study	
☐	☒	Antibodies
☒	☐	Eukaryotic cell lines
☒	☐	Palaeontology and archaeology
☒	☐	Animals and other organisms
☐	☒	Clinical data
☒	☐	Dual use research of concern
☒	☐	Plants

Methods

n/a	Involved in the study	
☒	☐	ChIP-seq
☐	☒	Flow cytometry
☒	☐	MRI-based neuroimaging

Antibodies

Antibodies used

Flow cytometry:
human anti-CD25 APC (clone BC96) 1:200 (BioLegend, #302610, Lot B455064)
human anti-CD69 APC/Cy7 (clone FN50) 1:200 (BioLegend, #310914, Lot B322246)
human anti-CD45 PB (clone HI30) 1:200 (BioLegend, #304029, Lot B360163)

Western blots:
human anti-β-actin (clone C4) 1:1000 (Santa Cruz, #sc-47778, Lot B0719)
human anti-c-Myc 1:1000 (Cell Signaling, #9402, Lot 12)
human anti-IκBα 1:1000 (Cell Signaling, #9242, Lot 10)
human anti-MAPK (Erk1/2) (clone 3A7) 1:1000 (Cell Signaling, #9107, Lot 10)
human anti-phospho-MAPK (Erk1/2) (T202/Y204) 1:1000 (Cell Signaling, #9101, Lot 30)
human anti-phospho-PLCγ1 (Y783) 1:1000 (Cell Signaling, #2821, Lot 9)
human anti-phospho-Zap-70 (Y319) (clone 65E4) 1:1000 (Cell Signaling, #2717, Lot 15)
human anti-PLCγ1 (clone D9H10) 1:1000 (Cell Signaling, #5690, Lot 1)
human anti-Zap-70 (clone D1C10E) 1:1000 (Cell Signaling, #3165, Lot 1)
polyclonal anti-mouse IgG 1:5000 (Jackson ImmunoResearch, #715-036-150, Lot 163870)
polyclonal anti-rabbit IgG 1:5000 (Jackson ImmunoResearch, #711-035-152, Lot 164222)

Validation

As all antibodies are commercially available products, information on relevant citations and antibody profiles in online databases can be accessed via the manufacturers website using the respective catalogue number given in the antibody list above.

Clinical data

Policy information about clinical studies
All manuscripts should comply with the ICMJE guidelines for publication of clinical research and a completed CONSORT checklist must be included with all submissions.

Clinical trial registration	not applicable
Study protocol	not applicable
Data collection	Retrospective collection of descriptive clinical data with patient consent.
Outcomes	not applicable

Plants

Seed stocks	not applicable
Novel plant genotypes	not applicable
Authentication	not applicable

Flow Cytometry

Plots

Confirm that:

☒ The axis labels state the marker and fluorochrome used (e.g. CD4-FITC).

☒ The axis scales are clearly visible. Include numbers along axes only for bottom left plot of group (a 'group' is an analysis of identical markers).

☒ All plots are contour plots with outliers or pseudocolor plots.

☒ A numerical value for number of cells or percentage (with statistics) is provided.

Methodology

Sample preparation	Peripheral blood mononuclear cells (PBMCs) of patient pair_1 were isolated via density gradient centrifugation (Histopaque, Sigma-Aldrich) and cryo-preserved. For TCR activation in vitro, cryo-preserved patient pair_1 PBMCs were thawed, incubated overnight, and adjusted to 2x10^6 PBMC/mL followed by stimulation using plate-bound human anti-CD3 (1 µg/mL) and human anti-CD28 (2 µg/mL) antibodies (OKT3/CD28.2, BioLegend). To analyze surface expression of T-cell activation markers, 2x10^5 PBMCs were stained 1:200 with fluorochrome-conjugated antibodies (supplemental Table 5).
Instrument	Surface marker expression was measured using a Beckman Coulter Gallios 3L 10C flow cytometer, instance number 1095673, serial number AN17052.
Software	Flow cytometry data was analyzed using the Beckman Coulter Kaluza software version 2.2.00000.20164, perpetual license type, serial number 251988913.
Cell population abundance	The abundance of T-PLL cells in the patient pair_1 indolent-stage and the active-stage samples were 94% and 96%, respectively, which was determined via FS/SS gating (supplemental Figures 10-11).
Gating strategy	1) FS/SS plots were used for gating of T-PLL cells. 2) Gated T-PLL cells were separated into CD45- (MFI<1.5) and CD45+ T-PLL cells (MFI>1.5) plotting SS against CD45 PB. 3) Both CD45- and CD45+ T-PLL cells were separatly analyzed regarding CD25 and CD69 expression via SS/CD25 APC and SS/CD69 APC/Cy7 plots, respectively. Here, CD25 MFI>10 defined CD25+ T-PLL cells and CD69 MFI>2 defined CD69+ T-PLL cells.

☒ Tick this box to confirm that a figure exemplifying the gating strategy is provided in the Supplementary Information.
